# Genetic characterization and pathogenicity of H7N9 highly pathogenic avian influenza viruses isolated from South China in 2017

**DOI:** 10.3389/fmicb.2023.1105529

**Published:** 2023-03-07

**Authors:** Bingbing Zhao, Wenqing Wang, Yating Song, Xiangyang Wen, Siyu Feng, Weiqiang Li, Yangbao Ding, Zuxian Chen, Zhuoliang He, Shao Wang, Peirong Jiao

**Affiliations:** ^1^Guangdong Laboratory for Lingnan Modern Agriculture, College of Veterinary Medicine, South China Agricultural University, Guangzhou, China; ^2^Guangdong Provincial Key Laboratory of Zoonosis Prevention and Control, Guangzhou, China; ^3^Animal Influenza Laboratory of the Ministry of Agriculture and Rural Affairs, Harbin Veterinary Research Institute, Chinese Academy of Agricultural Sciences, Harbin, China; ^4^Institute of Animal Husbandry and Veterinary Medicine, Fujian Academy of Agriculture Sciences, Fuzhou, China

**Keywords:** H7N9 highly pathogenic avian influenza virus, genetic characterization, pathogenicity, transmission, immune responses, chickens

## Abstract

Since 2017, the new H7N9 highly pathogenic avian influenza viruses (HPAIVs) have been responsible for more than 200,000 cases of chicken infection and more than 120,000 chicken deaths in China. Our previous study found that the Q26 was chicken-origin H7N9 HPAIV. In this study, we analyzed the genetic characterization of Q24, Q65, Q66, Q85, and Q102 H7N9 avian influenza viruses isolated from Guangdong, China in 2017. Our results showed that these viruses were highly pathogenic and belonged to two different genotypes, which suggested they occurred genetic reassortant. To investigate the pathogenicity, transmission, and host immune responses of H7N9 virus in chickens, we selected Q24 and Q26 viruses to inoculate chickens. The Q24 and Q26 viruses killed all inoculated chickens within 3 days and replicated effectively in all tested tissues. They were efficiently transmitted to contact chickens and killed them within 4 days through direct contact. Furthermore, we found that the expressions of several immune-related genes (e.g., TLR3, TLR7, MDA5, MAVS, IFN-β, IL-6, IL-8, OAS, Mx1, MHC I, and MHC II) were upregulated obviously in the lungs and spleen of chickens inoculated with the two H7N9 viruses at 24 h post-inoculation (HPI). Among these, IL-6 and IFN-β in lungs were the most upregulated (by 341.02–381.48-fold and 472.50–500.56-fold, respectively). These results suggest that the new H7N9 viruses isolated in 2017, can replicate and transmit effectively and trigger strong immune responses in chickens, which helps us understand the genetic and pathogenic variations of H7N9 HPAIVs in China.

## 1. Introduction

Avian influenza is a kind of zoonosis caused by avian influenza viruses (AIVs), which can infect host species, including wild and domestic birds, horses, pigs, marine mammals, and humans (Yoon et al., 2014). According to their different pathogenic abilities for poultry, AIVs can be divided into HPAIVs and low pathogenic avian influenza viruses (LPAIVs). HPAIVs are composed of some but not all H5 and H7 subtypes and can cause high mortality of birds. By contrast, LPAIVs generally cause much milder, primarily respiratory diseases (Alexander, 2000). It is possible that an H5 or H7 LPAIV mutates into HPAIV when several basic amino acids are inserted into its hemagglutinin cleavage site (Webster and Rott, 1987).

Since 2013, the newly merged H7N9 virus has caused 615 deaths in 1,568 laboratory-confirmed clinical cases worldwide (FAO, 2019). Based on the human infections of H7N9 virus in China, the virus was clustered into the Yangtze River Delta lineage and the Pearl River Delta lineage (Wang et al., 2016). Epidemiology investigations confirmed that virus-infected poultry and contaminated environment are the major sources of human infection with the H7N9 virus (Chen et al., 2013; Shi et al., 2013). Since 2017, some low pathogenic H7N9 viruses have mutated into highly pathogenic strains that have spread in chickens in 11 provinces of China and caused 200,196 cases of chicken infection with 128,563 deaths; a total of 1,074,648 chickens have been destroyed up to now (Ministry of Agriculture of People's Republic of China, 2017, 2018; Shi et al., 2017; Zeng et al., 2018). So, the new mutant H7N9 HPAIVs pose an increased threat to the chicken industry. However, the pathogenicity and transmission mechanisms of chickens infected with the new H7N9 HPAIVs have not been fully explored.

After AIVs infection, host innate and adaptive immune responses would be activated to induce the expressions of various cytokines, including PRRs, chemokines, ISGs, and the like, which could disturb the virus propagation process (Iwasaki and Pillai, 2014). It has been shown that host immune genes play an important role in the pathogenesis of HPAIV infection in chickens (Ranaware et al., 2016). Our previous study has also demonstrated that the expressions of host immune-related genes in ducks were associated with H5N1 HPAIVs infection (Wei et al., 2013). Furthermore, antiviral cytokines and virus-specific antibodies participated in the host immune response of chickens infected with early H7N9 LPAIVs in 2013 (Jiao et al., 2018). However, the host immune responses of chickens infected with the new H7N9 HPAIVs in 2017 is still unknown.

In our study, we analyzed the genetic evolution of five new H7N9 HPAIVs isolated from southern China in 2017, and selected the Q24 and Q26 viruses to study the pathogenicity, transmission, and host immune responses of them in chickens.

## 2. Materials and methods

### 2.1. H7N9 AIVs isolation and propagation

Five strains of new H7N9 AIVs, including A/chicken/Guangdong/Q24/2017 (named as Q24), A/chicken/Guangdong/Q65/2017 (Q65), A/chicken/Guangdong/Q66/2017 (Q66), A/chicken/Guangdong/Q85/2017 (Q85), and A/chicken/Guangdong/Q102/2017 (Q102) were isolated from tracheal and cloacal swabs of health chickens in live poultry markets in Guangdong, 2017. The isolation of A/chicken/Guangdong/Q26/2017 (Q26) was described in our previous study (Wang et al., 2017). These viruses were propagated and titrated in the allantoic cavities of 10-day-old specific-pathogen-free (SPF) chicken embryonated eggs at 37°C for 48 h. Virus titers were determined by inoculating 0.1 ml of 10-fold dilutions of the virus into the allantoic cavities and then calculating the 50% egg infectious dose (EID_50_) according to the method of Reed and Muench. All experiments involving live H7N9 AIVs were carried out in Animal Biosafety Level 3 (ABSL-3) facilities at South China Agricultural University.

### 2.2. Genetic characteristics

The extraction of viral RNA and the performance of PCR were based on our previous study (Jiao et al., 2016). Eight genes of Q24, Q65, Q66, Q85, and Q102 AIVs were sequenced. And then, we complied the sequencing data by the SeqMan program of Lasergene 7 (DNASTAR, Inc.) and constructed their phylogenetic trees by using the Neighbor Joining method and the Maximum Composite Likelihood model with MEGA 5.2 software (Sinauer Associates, Inc., Sunderland, MA, United States). The phylogenetic trees of the Q26 had been reported in our previous study (Wang et al., 2017). Related parameters of the trees were based on our previous study (Wang et al., 2017). The nucleotide sequences of H7N9 viruses in our study are available from NCBI GenBank with the accession numbers OQ024903, OP716876–OP716883, OP717067–OP717074, and OP718194–OP718216.

### 2.3. Animal experiments

Thirty-seven specific-pathogen-free white leghorn chickens (6 weeks old) purchased from Guangdong Wens Dahuanong Biotechnology Co., Ltd. were allocated randomly into two groups of 17 animals each. Three uninfected chickens as the negative control were euthanized and their lungs and spleen were collected to judge the expression of immune-related genes at 12 and 24 days post-inoculation (dpi) when we perform infection experiment. These collected lungs and spleen were stored at −80°C until used. All intranasally inoculated animals (*n* = 12) were administered with 10^6^ EID_50_ of Q24 or Q26 AIV in a 0.1 ml volume. At 12 and 24 HPI, three live inoculated chickens in each group were euthanized, and their brain, lungs, spleen, trachea, intestine, kidneys, and liver were collected and stored at −80°C until used. The collected tissues (1 g per tissue) were homogenized by PBS containing ampicillin (1,000 U/ml) and penicillin (1,000 U/ml), and centrifuged at 10,000 × *g* to isolate supernatant fluids. Then, the supernatant fluids were serially diluted by a factor of 10 and inoculated 100 μl diluted samples into 10-day-old embryonated eggs, respectively, to test viral replication. The remaining six inoculated chickens in each group were observed daily for clinical symptoms and mortality until 14 dpi or until all of them died of viral infection. To measure viral transmission in each group, five contact chickens were housed with the inoculated chickens after the inoculated chickens were infected with H7N9 virus. These contact chickens were intranasally inoculated with 0.1 ml phosphate buffer saline. The transmission was tested by detecting virus titers in the lungs of death contact chickens.

### 2.4. Quantitative analysis of cytokines

To investigate immune responses of chickens inoculated with new H7N9 HPAIVs, the expressions of TLR3, TLR7, MDA5, MAVS, IL-6, IL-8, IFN-β, OAS, Mx1, MHC I, and MHC II in the lungs and spleen homogenates were quantitatively determined. Total RNA (2 μg) was extracted from the lungs and spleen of euthanized chickens using RNeasy Plus Mini Kits (Qiagen, Germany) according to manufacturers’ instructions and was reverse transcribed. The resulting cDNA was stored at −20°C until further use. The primers used for quantitative real-time PCR (qRT-PCR) were designed using Oligo 7 software (Molecular Biology Insights Inc., Cascade, CO, United States) and selected based on specificity determined by dissociation curves. The qRT-PCR was performed based on our previous study (Jiao et al., 2018).

### 2.5. Ethics statement

All experiments involving animals were performed in ABSL-3 facilities and experimental protocols (SCAUABSL2017-019) were approved by the biosafety committee of South China Agriculture University. Housing animals were conducted in accordance with guidelines of the experimental animal administration and ethics committee of South China Agricultural University (SCAUABSL2017-019).

### 2.6. Statistical analysis

The statistical analyses were performed using GraphPad Prism 7.0 software (GraphPad software Inc., San Diego, CA, United States). Statistical significance differences were determined by using Student’s *t*-test. *p* values of <0.05, 0.01, and 0.001 were considered significant (^*^p < 0.05), highly significant (^**^p < 0.01), and extremely significant (^***^ *p* < 0.001), respectively.

## 3. Results

### 3.1. Genetic characteristics of H7N9 AIVs

**Figure 1:**
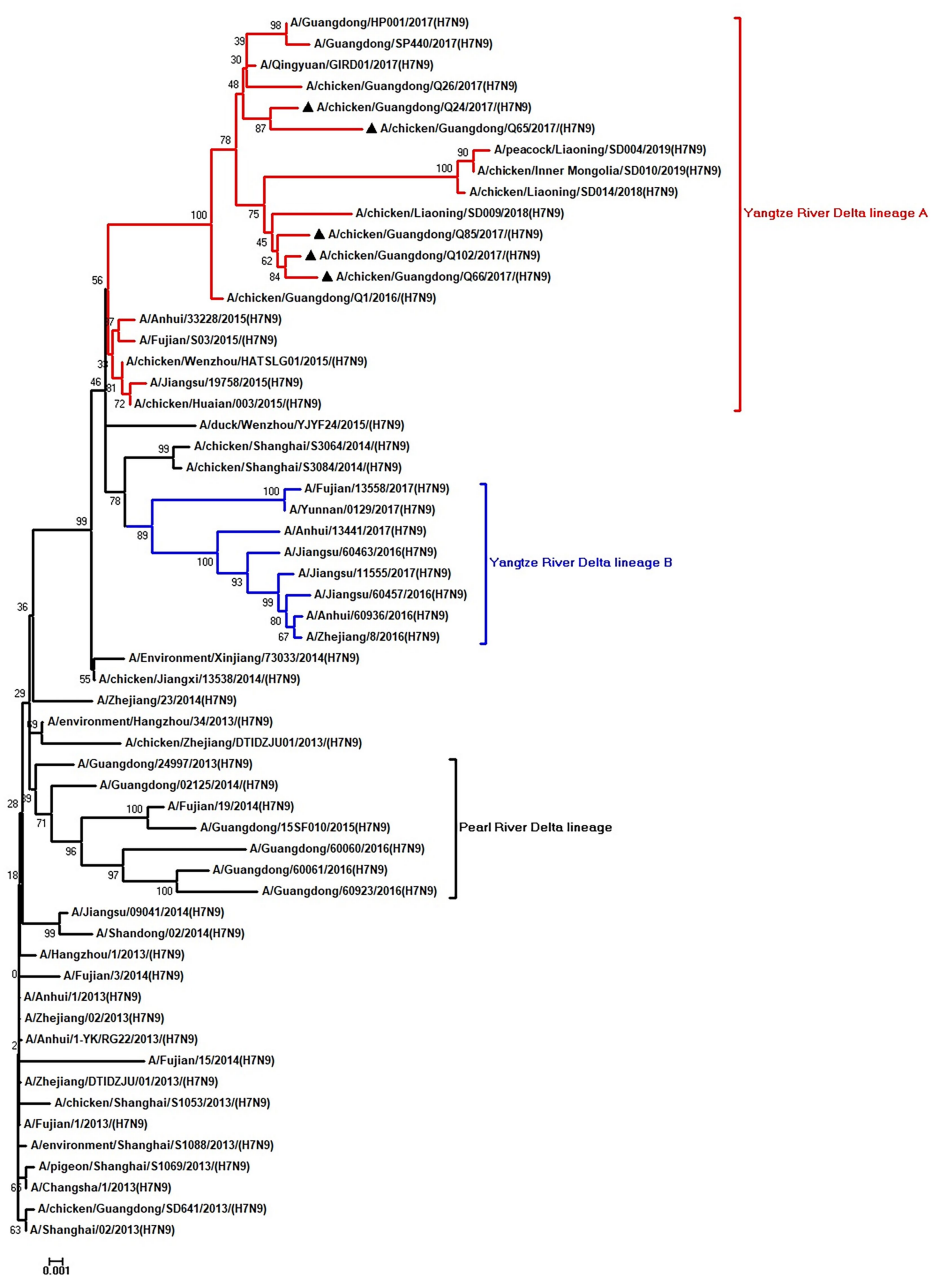
Phylogenetic analysis of HA. The tree was constructed by using the neighbor joining method with the Maximum Composite Likelihood model and MEGA version 5.2 (www.megasoftware.net/) with 1,000 bootstrap replicates based on the following sequences: HA: nucleotides (nt) 1–1,695. Except our five isolates, other virus sequences were downloaded from GenBank. Triangles indicate the viruses characterized in this study. The scale bar indicates the branch length and corresponds to 0.001 estimated amino acid substitutions per site.

**Figure 2:**
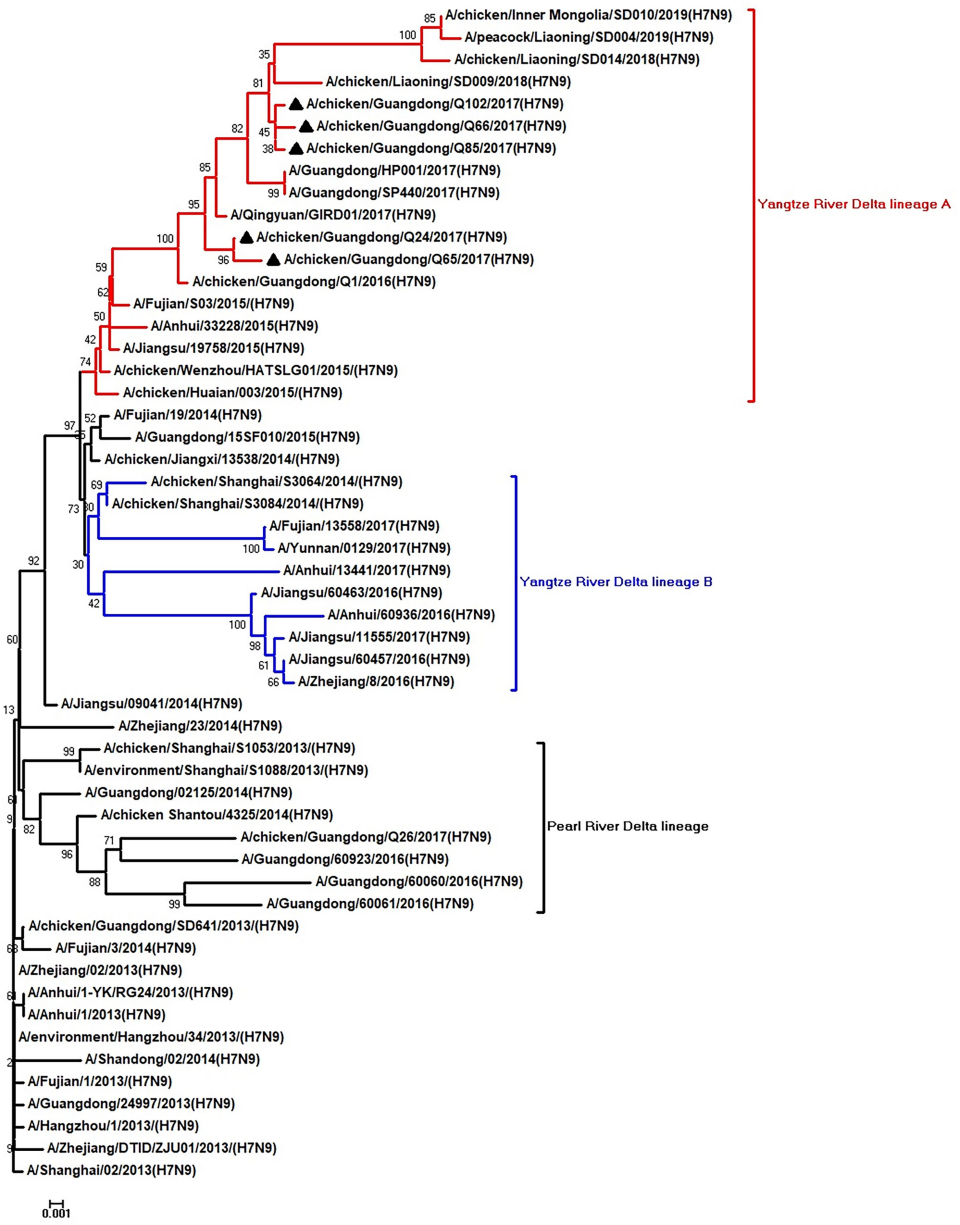
Phylogenetic analysis of NA. The tree was constructed by using the neighbor joining method with the Maximum Composite Likelihood model and MEGA version 5.2 (www.megasoftware.net/) with 1,000 bootstrap replicates based on the following sequences: NA: nt 19–1,416. Except our five isolates, other virus sequences were downloaded from GenBank. Triangles indicate the viruses characterized in this study. The scale bar indicates the branch length and corresponds to 0.001 estimated amino acid substitutions per site.

**Figure 3:**
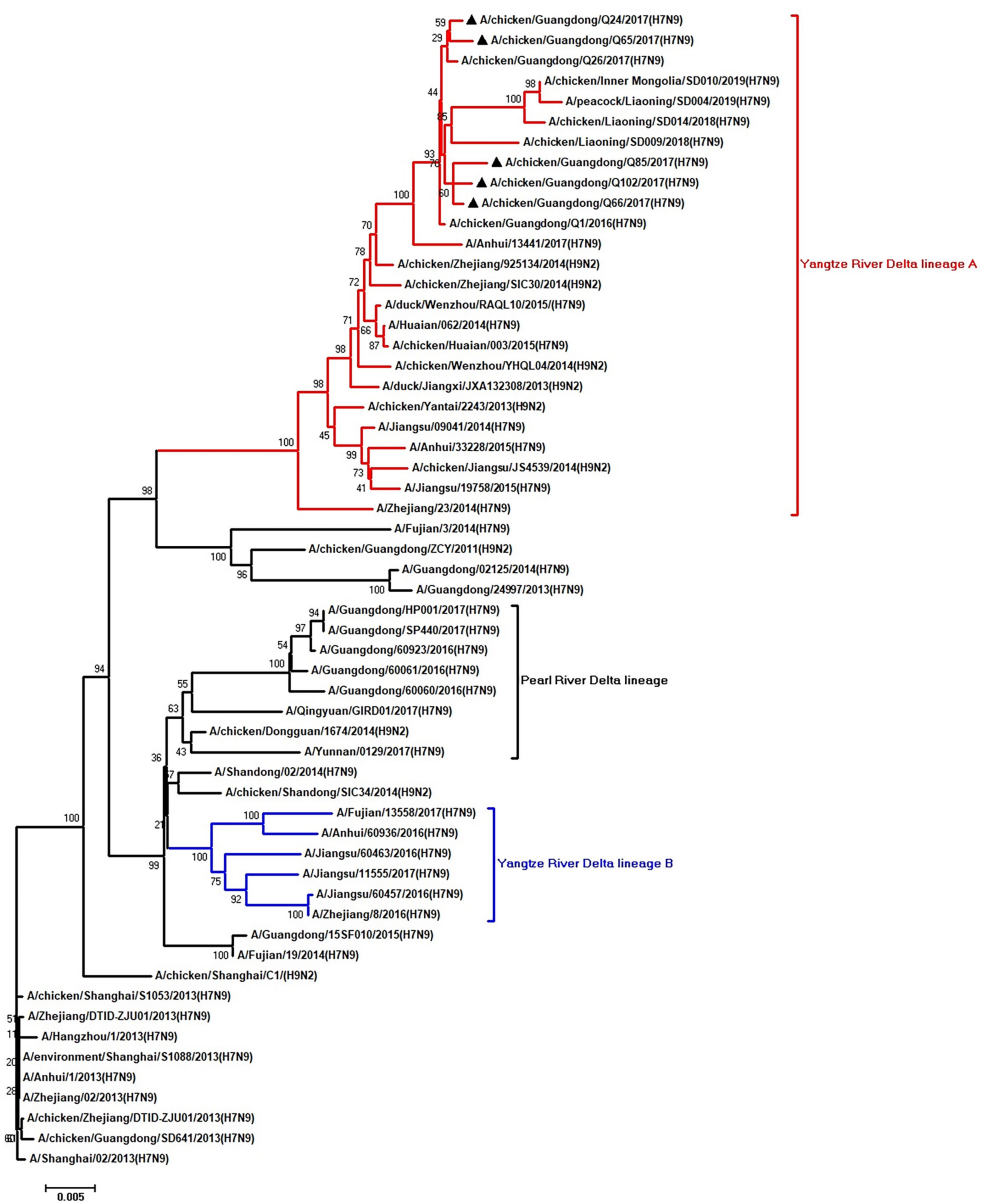
Phylogenetic analysis of PB2. The tree was constructed by using the neighbor joining method with the Maximum Composite Likelihood model and MEGA version 5.2 (www.megasoftware.net/) with 1,000 bootstrap replicates based on the following sequences: PB2: nt 28–2,307. Except our five isolates, other virus sequences were downloaded from GenBank. Triangles indicate the viruses characterized in this study. The scale bar indicates the branch length and corresponds to 0.002 estimated amino acid substitutions per site.

**Figure 4:**
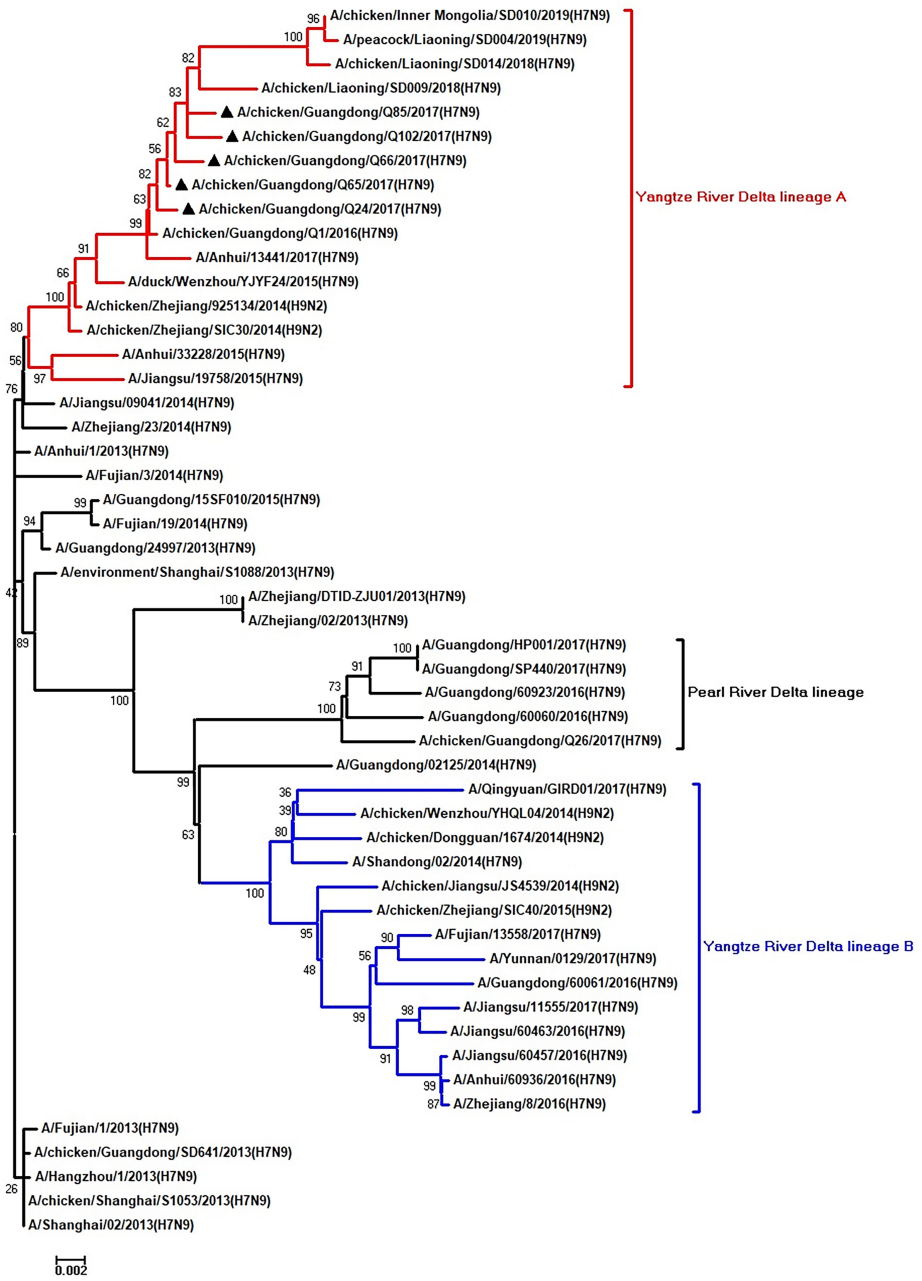
Phylogenetic analysis of PA. The tree was constructed by using the neighbor joining method with the Maximum Composite Likelihood model and MEGA version 5.2 (www.megasoftware.net/) with 1,000 bootstrap replicates based on the following sequences: PA: nt 25–2,175. Except our five isolates, other virus sequences were downloaded from GenBank. Triangles indicate the viruses characterized in this study. The scale bar indicates the branch length and corresponds to 0.002 estimated amino acid substitutions per site.

**Figure 5:**
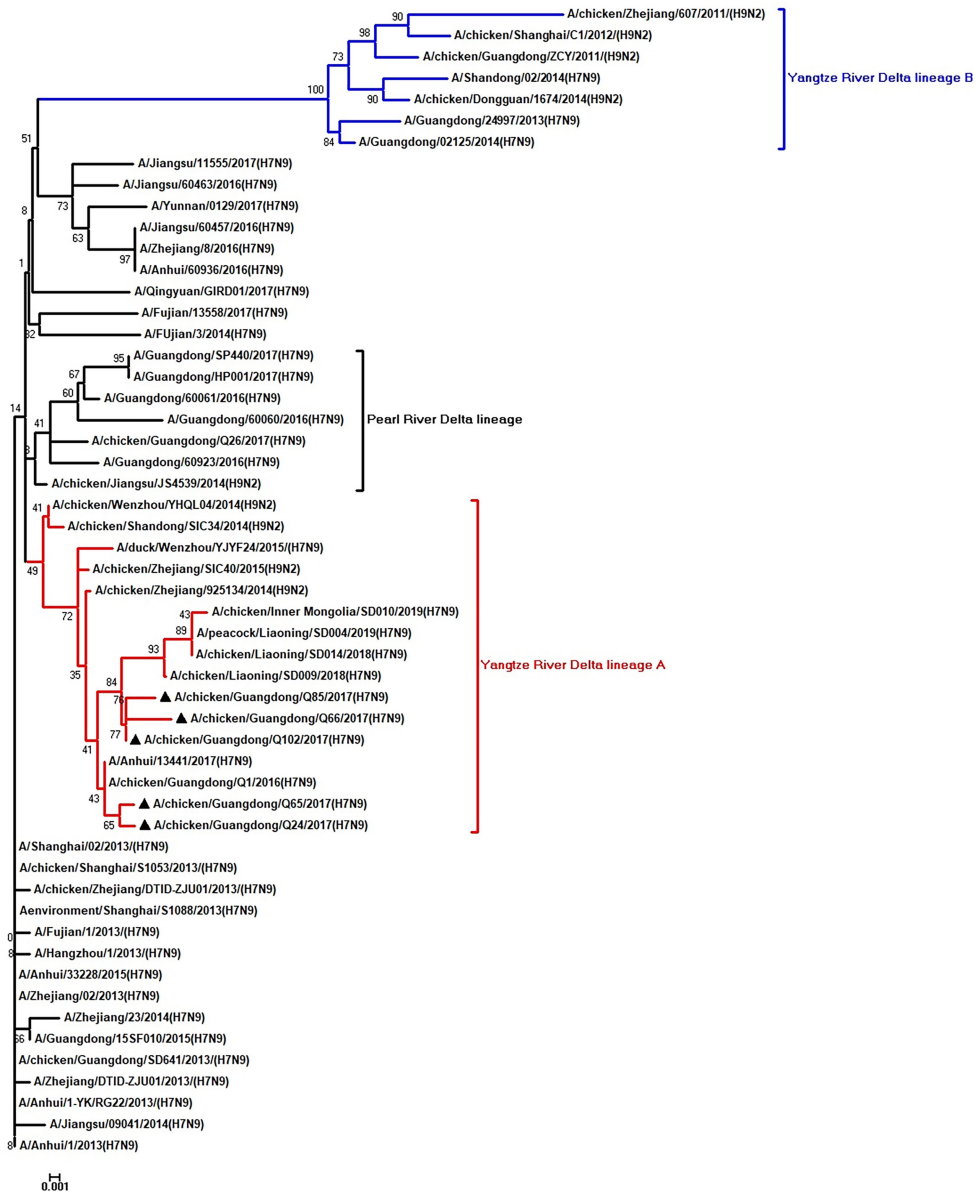
Phylogenetic analysis of NS. The tree was constructed by using the neighbor joining method with the Maximum Composite Likelihood model and MEGA version 5.2 (www.megasoftware.net/) with 1,000 bootstrap replicates based on the following sequences: NS: nt 27–680. Except our five isolates, other virus sequences were downloaded from GenBank. Triangles indicate the viruses characterized in this study. The scale bar indicates the branch length and corresponds to 0.001 estimated amino acid substitutions per site.

**Figure 6:**
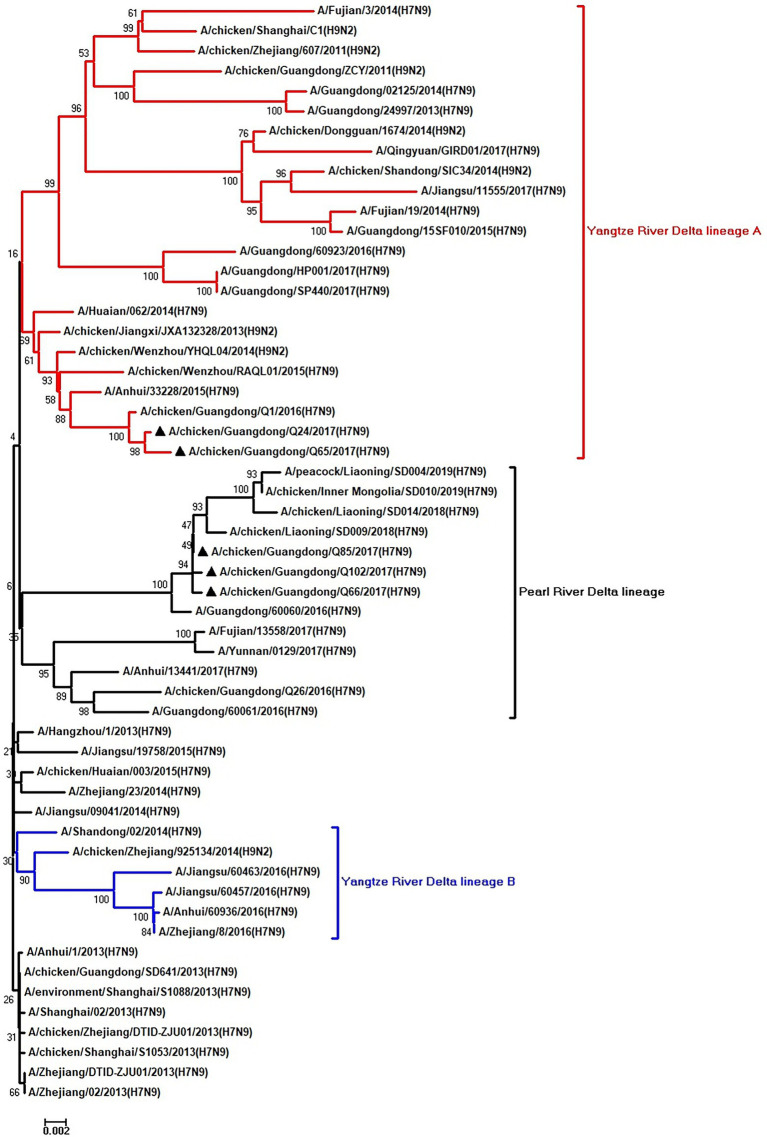
Phylogenetic analysis of PB1. The tree was constructed by using the neighbor joining method with the Maximum Composite Likelihood model and MEGA version 5.2 (www.megasoftware.net/) with 1,000 bootstrap replicates based on the following sequences: PB1: nt 25–2,298. Except our five isolates, other virus sequences were downloaded from GenBank. Triangles indicate the viruses characterized in this study. The scale bar indicates the branch length and corresponds to 0.002 estimated amino acid substitutions per site.

**Figure 7:**
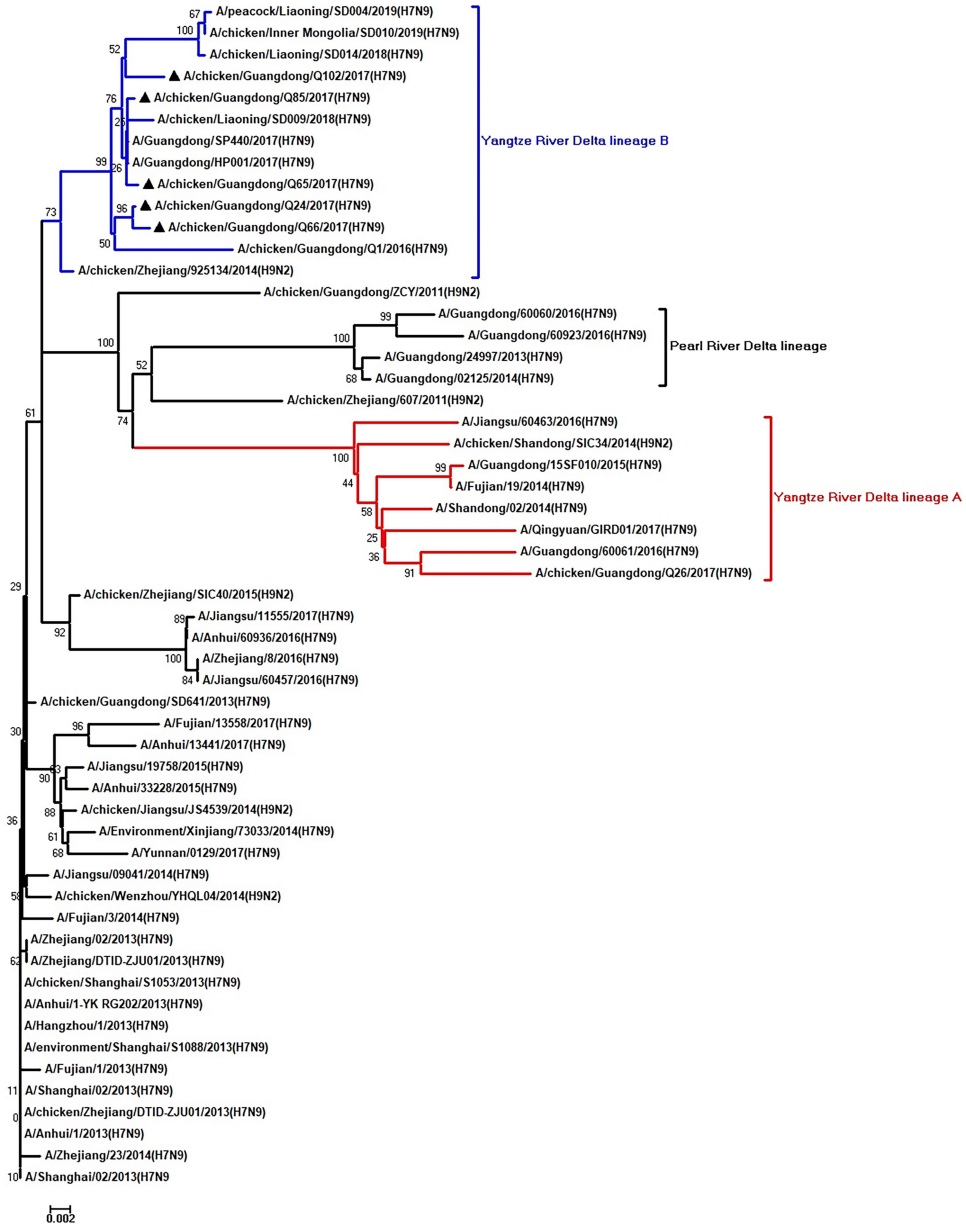
Phylogenetic analysis of NP. The tree was constructed by using the neighbor joining method with the Maximum Composite Likelihood model and MEGA version 5.2 (www.megasoftware.net/) with 1,000 bootstrap replicates based on the following sequences: NP: nt 46–1,542. Except our five isolates, other virus sequences were downloaded from GenBank. Triangles indicate the viruses characterized in this study. The scale bar indicates the branch length and corresponds to 0.002 estimated amino acid substitutions per site.

**Figure 8:**
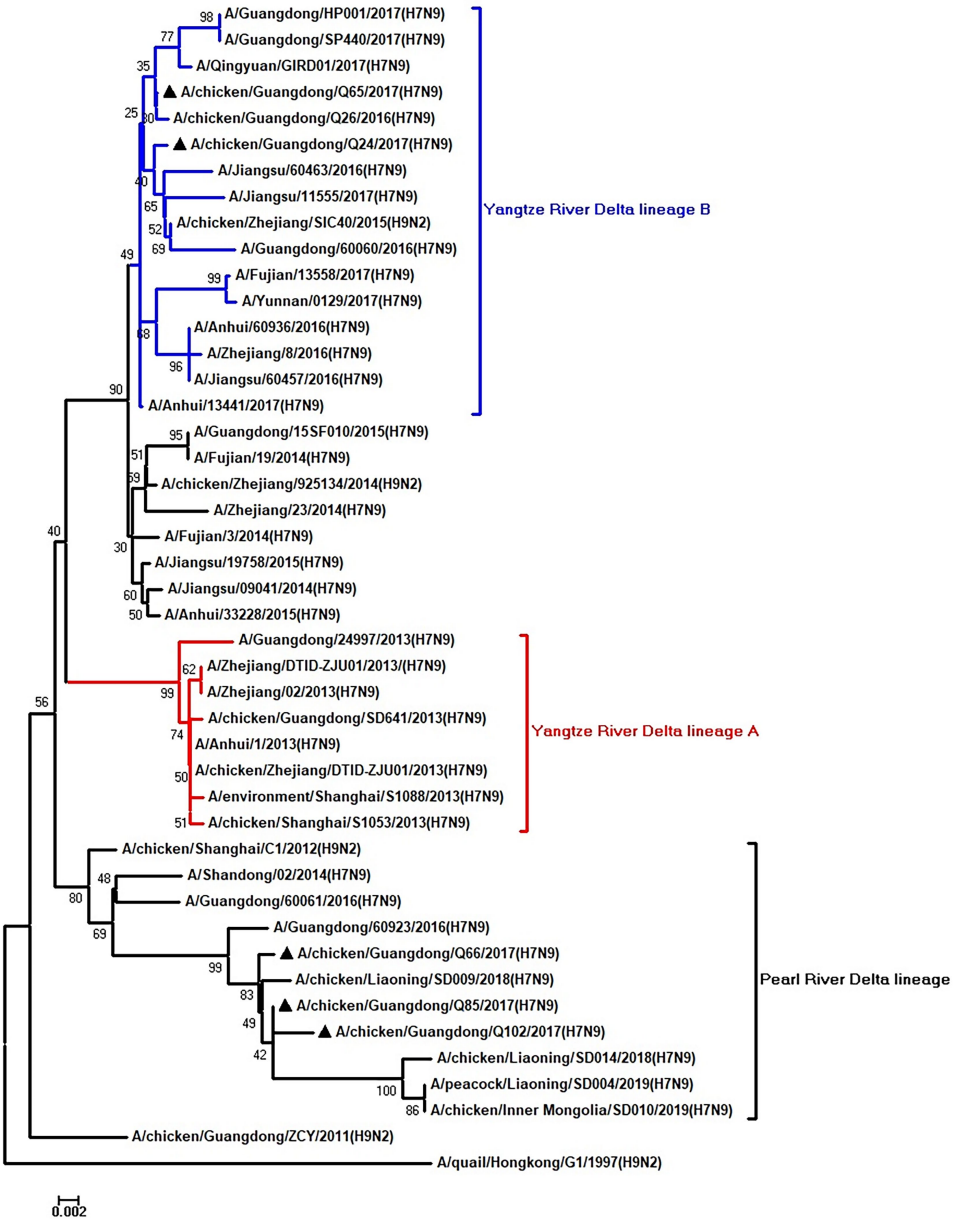
Phylogenetic analysis of M. The tree was constructed by using the neighbor joining method with the Maximum Composite Likelihood model and MEGA version 5.2 (www.megasoftware.net/) with 1,000 bootstrap replicates based on the following sequences: M: nt 26–784. Except our five isolates, other virus sequences were downloaded from GenBank. Triangles indicate the viruses characterized in this study. The scale bar indicates the branch length and corresponds to 0.002 estimated amino acid substitutions per site.

To clarify the origins and genetic characteristics of the new H7N9 AIVs, we sequenced eight genes of the Q24, Q65, Q66, Q85, and Q102 viruses. We found that the hemagglutinin (HA), neuraminidase (NA), polymerase basic 2 (PB2), acidic polymerase (PA), and nonstructural (NS) genes of the five AIVs were clustered into the Yangtze River Delta lineage A (Figures 1–5; Table 1). The polymerase basic 1 (PB1) genes of Q24 and Q65 were included in the Yangtze River Delta lineage A, whereas of other three AIVs were included in the Pearl River Delta lineage (Figure 6; Table 1). The nucleoprotein (NP) genes of the five AIVs were classified into the Yangtze River Delta lineage B (Figure 7; Table 1). The matrix (M) gene of Q24 and Q65 viruses derived from the Yangtze River Delta lineage B, whereas other three AIVs were clustered into Pearl River Delta lineage (Figure 8; Table 1). Therefore, the five H7N9 AIVs from Guangdong province in southern China in 2017 were clustered into two different genotypes.

**Table 1:** Summary of origins and genetic reassortant of the Q24, Q65, Q66, Q85, and Q102 viruses.

Viruses	Genes								Genetic	Genetic reassortant
	HA	NA	PB2	PB1	PA	NP	M1	NS1		
Q24	YRD Lineage A	YRD Lineage A	YRD Lineage A	YRD Lineage A	YRD Lineage A	YRD Lineage B	YRD Lineage B	YRD Lineage A	group 1	Yes
Q65	YRD Lineage A	YRD Lineage A	YRD Lineage A	YRD Lineage A	YRD Lineage A	YRD Lineage B	YRD Lineage B	YRD Lineage A	group 1	Yes
Q66	YRD Lineage A	YRD Lineage A	YRD Lineage A	PRD Lineage	YRD Lineage A	YRD Lineage B	PRD Lineage	YRD Lineage A	group 2	Yes
Q85	YRD Lineage A	YRD Lineage A	YRD Lineage A	PRD Lineage	YRD Lineage A	YRD Lineage B	PRD Lineage	YRD Lineage A	group 2	Yes
Q102	YRD Lineage A	YRD Lineage A	YRD Lineage A	PRD Lineage	YRD Lineage A	YRD Lineage B	PRD Lineage	YRD Lineage A	group 2	Yes

YRD Lineage A stands for the Yangtze River Delta lineage A; YRD Lineage B stands for the Yangtze River Delta lineage B; and PRD Lineage stands for the Pearl River Delta lineage.

The basic amino acids at the HA cleavage site of Q24 and Q65 viruses were “PEVPKGKRTARG,” and of other three viruses were “PEVPKRKRTARG,” which were the characteristics of HPAIVs (Shi et al., 2018; Table 2). The HA gene receptor-binding sites of the five AIVs at positions 226 and 228 were glutamine (Q) and glycine (G), which indicates that these viruses have the specificity of binding the avian-type receptor (Ramos et al., 2013; Table 2). Five potential N-linked glycosylation sites at sites 30, 46, 249, 425, and 497 were detected in the HA genes of the five viruses (Table 3). The NA gene of the five viruses had a five-amino-acid deletion of residues 69–73 in the stalk region, which fitted with the characteristic of early human-origin H7N9 virus (Shi et al., 2013; Table 2). So, the deletion may be associated with increased their virulence in mammals. Their NA genes had seven conservative potential glycosylation sites at positions 42, 52, 63, 66, 82, 142, and 197 (Table 3). The amino acids at site 627 and 701 of their PB2 gene of these viruses were glutamic acid (E) and aspartic acid (D), which was the characteristic of avian-origin influenza virus (Table 2). The site 31 of M2 gene in the five viruses was Asparagine (N), which could increase the resistance of these viruses to amantadine and rimantadine (Bean et al., 1989; Lan et al., 2010; Table 2). The P42S mutation of NS1 gene in the five H7N9 viruses indicated they may have strong virulence for mice (Jiao et al., 2008; Table 2).

**Table 2:** Summary of signature amino acids in Q24, Q65, Q66, Q85, and Q102 viruses.

Genes^a^	Amino acid position	Viruses				
		Q24	Q65	Q66	Q85	Q102
HA	226	Q	Q	Q	Q	Q
	228	G	G	G	G	G
	333–346^b^ (Cleavage site)	PEVPKGKRTARG	PEVPKGKRTARG	PEVPKRKRTARG	PEVPKRKRTARG	PEVPKRKRTARG
NA	69–73^b^	deletion	deletion	deletion	deletion	deletion
PB2	627	E	E	E	E	E
	701	D	D	D	D	D
NP	286	A	A	A	A	A
	437	T	T	T	T	T
M2	31^b^	N	N	N	N	N
NS1	42^b^	S	S	S	S	S

^a^According to the genes of A/Anhui/1/2013 numbering; ^b^Amino acid sites occurred mutation.

**Table 3:** Summary of the potential glycosylation sites in HA and NA genes of the Q24, Q65, Q66, Q85, and Q102 viruses.

Genes^a^	Amino acid position	Viruses				
		Q24	Q65	Q66	Q85	Q102
HA	30	NGT	NGT	NGT	NGT	NGT
	46	NAT	NAT	NAT	NAT	NAT
	249	NDT	NDT	NDT	NDT	NDT
	425	NWT	NWT	NWT	NWT	NWT
	497	NNT	NNT	NNT	NNT	NNT
NA	42	NCS	NCS	NCS	NCS	NCS
	52	NTS	NTS	NTS	NTS	NTS
	63	NET	NET	NET	NET	NET
	66	NIT	NIT	NIT	NIT	NIT
	82	NLT	NLT	NLT	NLT	NLT
	142	NGT	NGT	NGT	NGT	NGT
	197	NAS	NAS	NAS	NAS	NAS

^a^According to the genes of A/Anhui/1/2013 numbering.

### 3.2. Pathogenicity, replication, and transmission of H7N9 AIVs in chickens

**Figure 9:**
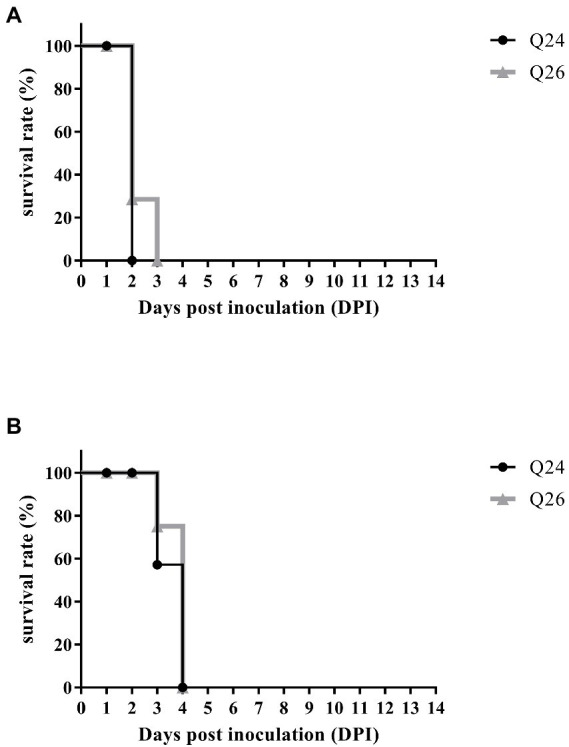
The percent survival of the inoculated and contact chickens. Six inoculated chickens in the Q24 or Q26 group were observed daily for clinical symptoms and mortality, respectively. Five contact chickens were housed with the inoculated chickens in each group, respectively, to measure viral transmission in chickens (direct contact transmission). The survival curves of **(A)** inoculated and **(B)** contact chickens were drawn basing on GraphPad Prism 7.0 software.

To examine the pathogenicity of new H7N9 AIVs in chickens, we intranasally inoculated birds with 10^6^ EID_50_ of the Q24 or Q26 H7N9 AIV in a 0.1 ml volume. We found that the chickens inoculated with Q24 and Q26 H7N9 AIVs started to show signs of typical clinical symptoms at 1 DPI, such as eyelid edema, torticollis, and insensibility. Chickens inoculated with the two viruses began dying at 2 dpi, and the remaining chickens all died within 3 dpi (Figure 9A). Thus, the Q24 and Q26 H7N9 AIVs were highly pathogenic to chicken.

To evaluate the replicabilities of the two H7N9 HPAIVs in chickens, three inoculated chickens in each group were euthanized at 12 and 24 HPI, respectively. Their brain, intestine, kidneys, liver, lungs, spleen, and trachea were harvested to test virus titers, respectively. In the Q24 and Q26 virus-inoculated chickens, the viruses were detected in all tested tissues at 12 and 24 HPI. Virus titers in the lungs were 3.42–4.0 log_10_ EID_50_ at 12 HPI and 6.25–8.08 log_10_ EID_50_ at 24 HPI. Virus titers in the brain, intestine, kidneys, liver, spleen, and trachea were 1.83–2.42, 2.17–2.75, 3.17–3.25, 3.08–3.5, 2.08–2.42, and 3.42–4.33 log_10_ EID_50_ at 12 HPI and 3.75–4.67, 5.67–6.42, 5.75–7.16, 5.5–5.67, 6.25–7.80, and 4.67–7.33 log_10_ EID_50_ at 24 HPI, respectively (Table 4). Overall, both the Q24 and Q26 H7N9 HPAIVs replicated effectively in all tested tissues at 12 and 24 HPI. In particular, these H7N9 HPAIVs could cross the blood–brain barrier to replicate in brains.

**Table 4:** Replication in the chickens of the H7N9 viruses after inoculated intranasally^a^.

Strains	Virus replication in tissues of infection chickens (log_10_ EID_50_/g/0.1 ml)^a^								Lungs of contact chickens
	Times	Liver	Spleen	Lungs	Kidney	Brain	Trachea	Intestine	(log_10_ EID_50_/g/0.1 ml)^a^
Q24	12 HPI	3.08 ± 0.29	2.08 ± 0.76	3.42 ± 0.29	3.25 ± 0.66	1.83 ± 0.38	3.42 ± 0.58	2.17 ± 0.38	8.9 ± 0.36
	24 HPI	5.50 ± 0	6.25 ± 0.50	6.25 ± 0.50	5.75 ± 0.50	3.75 ± 1.09	4.67 ± 0.14	5.67 ± 0.14	
Q26	12 HPI	3.50 ± 0.25	2.42 ± 0.29	4.0 ± 0.52	3.17 ± 1.16	2.42 ± 0.76	4.33 ± 0.52	2.75 ± 1.39	8.6 ± 0.42
	24 HPI	5.67 ± 0.52	7.80 ± 0.52	8.08 ± 0.58	7.16 ± 1.46	4.67 ± 0.14	7.33 ± 1.01	6.42 ± 0.76	

HPI, hours post-infection. ^a^The SPF chickens were assigned randomly to two groups and were inoculated intranasally (i.n.) with 10^6^ EID_50_ of Q24 and Q26 H7N9 avian influenza viruses in a volume of 100 μl, respectively. To compare the change of viruses in different time points, three chickens in every cage were euthanized at 12 and 24 HPI, and their titers were detected in tissues of brain, intestine, kidneys, liver, lungs, spleen, and trachea in eggs; ^b^A value of 1.5 was assigned if the virus was not detected from the undiluted samples in three embryonated hen eggs. Virus titers are expressed as means ± SD in log_10_ EID_50_/g/0.1 ml of tissue.

To assess the transmission of Q24 and Q26 H7N9 HPAIVs in chickens, five contact chickens were housed with the inoculated chickens in each group. The lungs of the dead contact chickens were collected to test virus titers during the experiment period. We found that the contact chickens in the Q24 and Q26 groups died within 4 dpi (Figure 9B). The mean titers of the two viruses in the lungs of the contact chickens were 8.9 and 8.6 log_10_ EID_50_, respectively (Table 4). So, the two H7N9 HPAIVs could be horizontally spread among chickens by contact and cause the deaths of all contact chickens.

### 3.3. Immune responses in chickens after inoculation with H7N9 AIVs

**Figure 10:**
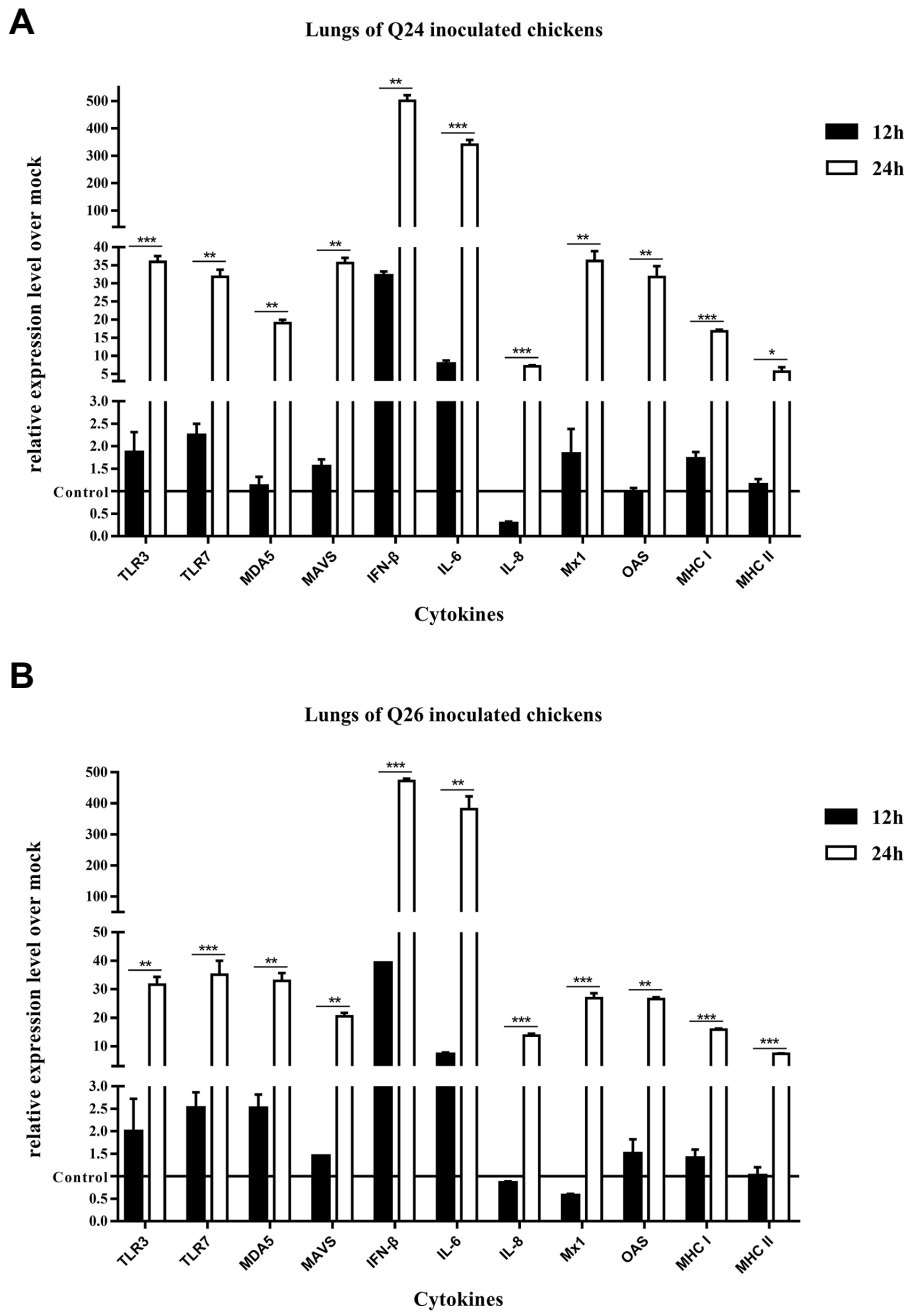
The expressions of immune-relate genes in the lungs of inoculated chickens. Comparing of the expressions of TLR3, TLR7, MDA5, MAVS, IFN-β, IL-6, IL-8, Mx1, OAS, MHC I, and MHC II in the lungs harvested from the inoculated chickens by **(A)** Q24 and **(B)** Q26 H7N9 AIVs at 12 and 24 HPI, respectively. They were tested using the quantitative RT-PCR and calculated as follows: fold change = 2^-△△Ct^, which △ = Ct target-Ct β-action, and △△Ct = △Ct treated-△Ct control. Therefore, the expressions of all of the cytokines in the health control chickens were 1. We carried out the Student’s *t*-test between the experimental groups at 12 and 24 HPI, *p* < 0.05 was identified statistically significant (^*^p < 0.05; ^**^p < 0.01; ^***^p < 0.001).

**Figure 11:**
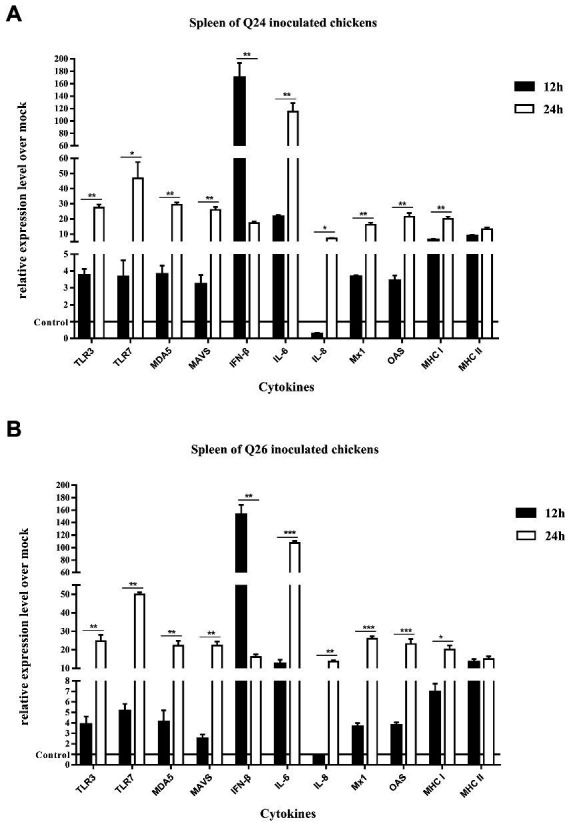
The expressions of immune-relate genes in the spleen of inoculated chickens. Comparing of the expressions of TLR3, TLR7, MDA5, MAVS, IFN-β, IL-6, IL-8, Mx1, OAS, MHC I, and MHC II in the spleen harvested from the inoculated chickens by **(A)** Q24 and **(B)** Q26 H7N9 AIVs at 12 and 24 HPI, respectively. They were tested using the quantitative RT-PCR and calculated as follows: fold change = 2^-△△Ct^, which △ = Ct target-Ct β-action, and △△Ct = △Ct treated-△Ct control. Therefore, the expressions of all of the cytokines in the health control chickens were 1. We carried out the Student’s *t*-test between the experimental groups at 12 and 24 HPI, *p* < 0.05 was identified statistically significant (^*^p < 0.05; ^**^p < 0.01; ^***^p < 0.001).

To observe the changes in PRRs and adaptor protein expression during the antiviral immune response of chickens inoculated with H7N9 HPAIVs, the productions of TLR3, TLR7, MDA5, and MAVS were detected by quantitative real-time PCR in the lungs and spleen of inoculated chickens at 12 and 24 HPI. In the lungs of Q24 and Q26 virus-inoculated chickens, their expressions hardly changed at 12 HPI, but were upregulated significantly (by 31.59–35.98, 31.84–35.17, 19.04–32.92, and 20.42–35.67-fold) at 24 HPI (*p* < 0.01), respectively (Figures 10A,B). In the spleen, their expressions were slightly increased (by 3.75–3.86, 3.65–5.13, 3.80–4.09, and 2.47–3.20-fold) at 12 HPI and clearly upregulated (by 24.67–27.41, 46.72–49.88, 22.18–29.35, and 22.39–25.89-fold) at 24 HPI (*p* < 0.05; Figures 11A,B). These data show that the expressions of PRRs and adaptor protein in the lungs and spleen of chickens inoculated with the two H7N9 HPAIVs were upregulated more significantly at 24 HPI than at 12 HPI.

To compare the expressions of cytokines in chickens inoculated with H7N9 HPAIVs, IL-6, IL-8, and IFN-β levels in the lungs and spleen of inoculated chickens were measured by quantitative real-time PCR at 12 and 24 HPI. In the lungs of Q24 and Q26 viruses-inoculated chickens, the expressions of IL-6, IL-8, and IFN-β were increased at 12 HPI (by 7.11–7.71, 0.27–0.85, and 32.04–39.10-fold) and at 24 HPI (341.02–381.48, 7.10–13.74, and 472.50–500.56-fold; *p* < 0.01; Figures 10A,B). In the spleen, their expressions were upregulated 12.49–21.65, 0.27–0.85, and 152.71–169.71-fold at 12 HPI, and 107.53–115.06, 6.51–9.91, and 16.15–17.16-fold at 24 HPI (*p* < 0.05), respectively (Figures 11A,B). Therefore, the expressions of IL-6 and IFN-β were significantly upregulated in the lungs and spleen of chickens inoculated with H7N9 HPAIVs at 12 and 24 HPI. However, the expression of IL-8 was hardly changed at 12 HPI, and only slightly upregulated at 24 HPI.

To confirm the expressions of antiviral molecules in chickens inoculated with H7N9 HPAIVs, we measured the myxovirus resistance protein 1 (Mx1) and 2′,5′-oligoadenylate synthetase (OAS) in the lungs and spleen of chickens inoculated with Q24 and Q26 HPAIVs by quantitative real-time PCR at 12 and 24 HPI, respectively. In the lungs of Q24 and Q26 viruses-inoculated chickens, their expressions were obviously upregulated by 26.90–36.22 and 26.56–31.78-fold at 24 HPI (*p* < 0.01), respectively (Figures 10A,B). In the spleen, their expressions were slightly increased (by 3.64–3.66 and 3.42–3.78-fold) at 12 HPI and significantly upregulated (by 16.20–26.05 and 21.51–23.17-fold) at 24 HPI (*p* < 0.01), respectively (Figures 11A,B). These data show that the expressions of Mx1 and OAS in the lungs and spleen of chickens inoculated with the two H7N9 HPAIVs were clearly upregulated at 24 HPI.

To investigate the changes in MHC I and MHC II expression in chickens inoculated with H7N9 HPAIVs, we measured their mRNA levels in the lungs and spleen of chickens inoculated with Q24 and Q26 H7N9 HPAIVs at 12 and 24 HPI using quantitative real-time PCR. In the lungs of Q24 and Q26 virus-inoculated chickens, their expressions hardly changed at 12 HPI, but were upregulated (by 15.84–16.71 and 5.63–7.38-fold, respectively) at 24 HPI (*p* < 0.05; Figures 10A,B). In the spleen, their expressions were upregulated significantly at 12 HPI (by 6.26–6.93 and 8.80–13.33-fold, respectively) and at 24 HPI (by 20.09–20.19 and 13.19–15.0-fold; Figures 11A,B). These results show that the expressions of MHC I and MHC II in the lungs and spleen of chickens inoculated with the two H7N9 HPAIVs were clearly upregulated at 24 HPI.

## 4. Discussion

In 2013, the novel H7N9 low pathogenic influenza A virus (LPIAV) was firstly isolated from three cases of human infection and death in Shanghai or Anhui Province, China (Gao et al., 2013; Zhang et al., 2013). Gao et al. (2013) analyzed the three human-origin H7N9 LPIAVs and found that these viruses were triple reassortant viruses, whose HA gene shared the highest identity with A/duck/Zhejiang/12/2011 (H7N3, subtype ZJ12), NA gene shared the highest identity with A/wild bird/Korea/A14/2011 (H7N9, subtype KO14), and other six internal genes were most closely related to A/brambling/Beijing/16/2012-like viruses (H9N2). Although the early H7N9 viruses could be isolated in chickens, no signs of typical clinical symptoms were seen in chickens, and no any avian species were caused disease (Shi et al., 2013; Zhang et al., 2013). Since 2013, the H7N9 virus had caused five waves of human infection and 615 deaths in the 1,568 laboratory-confirmed clinical cases in the whole world (WHO, 2019). Wang et al. analyzed 232 H7N9 viruses in early three waves and found that HA gene of viruses in wave 1 originated exclusively from the Yangtze River Delta region and transmitted in all affected regions, and clade W2-1 viruses emerging during wave 2 originated from the Pearl River Delta region and only transmitted in Guangdong, Guangxi, and Hunan provinces (Wang et al., 2016). In addition, they found that several viruses in clade W2-4 emerging during wave 2, which originated from the Yangtze River Delta region, were repeatedly introduced into the Pearl River Delta region and isolated in Guangdong (Wang et al., 2016). Between July 2013 and January 2017, Shi et al. divided 83 H7N9 viruses collected from poultry markets, farms, wild bird habitats, and slaughter houses in 24 provinces of China into 23 (G1–G23) genotypes basing on the gene identity from the phylogenetic analysis, and found that the G1 and G2 were the predominant genotype and could be detected in eight and nine provinces, respectively (Shi et al., 2017). In late 2016, the HA gene of low pathogenic H7N9 virus was inserted by several basic amino acids and mutated into highly pathogenic strains, which could infect humans, meanwhile, cause disease outbreaks in chickens (Wang et al., 2017). The H7N9 HPAIVs had caused more than 200,000 cases of chicken infection and more than 120,000 chicken deaths in China (Ministry of Agriculture of People's Republic of China, 2017, 2018). Between February 2017 and January 2018, Shi et al. isolated 81 H7N9 HPAIVs in 23 provinces of China and divided them into nine (G1-G9) genotypes, and found that the G2 was the predominant genotype and could be detected in 13 provinces (Shi et al., 2018). Between February 2018 and December 2019, Yin et al. isolated 19 H7N9 HPAIVs from two provinces in southern China (Anhui and Fujian) and six provinces in northern China (Shaanxi, Shanxi, Inner Mongolia, Ningxia, Hebei, and Liaoning). Around 18 of the 19 viruses belong to the genotype 2, whereas DK/FJ/SE0377/18 formed a different genotype, which was assigned to genotype 10 (G10; Yin et al., 2021). In our study, the five chicken-origin H7N9 viruses from Guangdong province in 2017 were divided into two genotypes (group 1 and group 2), which suggests that these viruses occur genetic reassortant. So, it is necessary to strengthen the surveillance of H7N9 AIVs in southern China.

The LPIAVs mutate into highly pathogenic strains through inserting several basic amino acids in their hemagglutinin cleavage site. Previous study found five different motifs in the HA cleavage sites of H7N9 HPAIVs: -KGKRTAR/G-, -KRKRAAR/G-, -KRKRTAR/G-, -KGKRIAR/G-, and -KRRRTAR/G- (Shi et al., 2017). In our study, the five H7N9 HPAIVs have two kinds of motifs. The HA cleavage sites (“PEVPKGKRTARG”) of Q24 and Q65 viruses were same with the motif -KGKRTAR/G-, which only isolated from birds-origin virus. The HA cleavage sites (“PEVPKRKRTARG”) of Q26, Q66, Q85, and Q102 viruses were same with the motif -KRKRTAR/G-, which could be isolated from birds-origin and humans-origin viruses. Therefore, the Q66, Q85, and Q102 H7N9 HPAIVs may be pathogenic to humans. Substitution Q226L at the 210-loop of HA gene was also detected in the early environment, human, and birds-origin H7N9 viruses in 2013, and in the chicken-origin H7N9 HPAIV in 2017, increasing the human receptor binding of the viruses (Shi et al., 2013; Wang et al., 2017). Zhu et al. found that although a few H7N9 HPAIVs still showed typical dual receptor preference, 226 L had mutated back to 226Q in their HA gene (Zhu et al., 2017). In our study, the site 226 of HA gene in our five H7N9 HPAIVs was glutamine (Q). Other mutations, 627 K in PB2 and 42S in NS1 of H7N9 LPIAVs in 2013, were also identified, which increased viral pathogenicity in mice (Gao et al., 2013). Wang et al. isolated 146 H7N9 LPIAVs in China in 2013 and found that the site 627 of PB2 segments in 58 non-human viruses and 24 human viruses were glutamic acid (E), and in other 1 non-human virus and 24 human viruses were lysine (K; Wang et al., 2014). In our study, the mutation of E627K in PB2 gene was not appeared, but the P42S mutation of NS1 gene in our five viruses was detected, which was related to inhibiting the production of interferon and increasing their virulence for mice (Jiao et al., 2008). Ma et al. found that the mutations of A286V and T437M in NP gene of H7N9 HPAIVs isolated in 2017 could attenuated their virulence in mice through inhibited the process of NP import to and export from the nucleus, which were not detected in our five viruses (Ma et al., 2020). Furthermore, the S31N mutation of M2 gene in our five H7N9 viruses indicated that they were not sensitive to the drugs (Bean et al., 1989; Lan et al., 2010). It is worth noting that these mutations of different amino acids in the H7N9 HPAIVs from Guangdong in 2017 may improve their adaptive capacity and virulence to human, mammal, and poultry hosts.

In 2013, the H7N9 virus from birds was a low pathogenic influenza virus that could infect chickens but not cause death (Zhang et al., 2013). The human-origin H7N9 virus (A/Anhui/1/13) replicated only in the trachea of the inoculated chickens at 2 DPI and in the lungs at 3 and 5 DPI (Ku et al., 2014). Compared with human-origin H7N9, our previous study showed that two chicken-origin H7N9 LPAIVs in 2013 replicated in several detected tissues of the chickens infected with a large dose of 10^8^ EID_50_ viruses in 0.2 ml volume (Jiao et al., 2018). So, it is clear that H7N9 strains in 2013 showed different replicabilities in chickens. Since 2017, some H7N9 LPAIVs began inserting several amino acids at the cleavage site of their hemagglutinin to mutate into HPAIVs, which had been detected in the samples collected from live poultry markets in Guangdong (Shi et al., 2018). Next, these H7N9 HPAIVs had caused 14 outbreaks in chicken farms of China, and had resulted in higher mortality rates among infected chickens (Ministry of Agriculture of People's Republic of China, 2017, 2018). Chicken-origin H7N9 HPAIV caused the death of all infected chickens within 24 HPI, and viral titers were 5.3–7.9 log_10_ EID_50_ in all tested tissues (Shi et al., 2017). We also found that all chickens inoculated with chicken-origin H7N9 HPAIVs died within 4 dpi, and the mean titers in tested tissues were 4.92–8.42 log_10_ EID_50_ at 3 dpi (Wang et al., 2017). Because the hemagglutinin cleavage sites of Q24 and Q26 viruses are different, which likely affect the pathogenicity and transmission of influenza virus. So, we chose the Q24 and Q26 viruses for animal experiments and found that the mortality rates of the chickens inoculated with chicken-origin Q24 and Q26 H7N9 HPAIVs were 100%. The two viruses replicated efficiently in all tested tissues. Therefore, the new chicken-origin H7N9 HPAIVs in 2017 have higher fatality rates and stronger replicabilities in chickens than those viruses isolated in 2013, which remains a serious threat to the poultry industry.

H7N9 influenza virus could be carried by various species and spread to other animals by aerosol, droplets, and contact (Zhang et al., 2013; Luk et al., 2015). In March 2017, H7N9 HPAIVs first caused a severe outbreak in chickens in Hunan. Within 14 months, highly pathogenic H7N9 avian influenza pandemics in chickens continuously occurred in 11 provinces of China. H7N9 HPAIVs mutated from H7N9 LPAIVs could transmit efficiently among chickens and cause serious damage to the chicken industry. Our previous study has shown that three chicken-origin H7N9 HPAIVs were transmitted from inoculated chickens to contact chickens and caused the deaths of contact chickens within 7 dpi, and their titers in all tested tissues of contact chickens were 4.92–8.42 log_10_ EID_50_ at 3 dpi (Wang et al., 2017). In this study, the mortality rates of contact chickens in the Q24 and Q26 groups were 100%, and mean virus titers in their lungs were 8.9 and 8.6 log_10_ EID_50_, respectively. Therefore, the new H7N9 HPAIVs could adapt well and transmit efficiently in chickens, which is significantly different from the early human-origin H7N9 virus in 2013.

When influenza virus infects host cells, the host PRRs (such as TLR3, TLR7, and MDA5) can be activated by viral RNA to trigger the expressions of transcription factors and the production of antiviral genes, such as interferons, which could stimulate the expressions of hundreds of IFN-stimulated genes (ISGs) in adjacent cells to induce antiviral innate immune response, and thus inhibit virus replication (Iwasaki and Pillai, 2014). Like in mammalian, several PRRs exist in chickens, such as TLR3, TLR7, and MDA5, which could also be activated by viral RNA to generate the antiviral immune responses during virus infection (Barjesteh et al., 2016). The previous study found that the expressions of TLR3, TLR7, and MDA5 were increased obviously in the lungs of chickens infected with chicken-origin H7N1 LPAIV at 1 dpi, and in the brains of chickens infected with chicken-origin H7N9 LPAIV at 3 dpi (Cornelissen et al., 2012; Jiao et al., 2018). Furthermore, the expressions of TLR3 and MDA5 in the lungs, brain, and spleen of chickens infected by turkey-origin H7N1 HPAIV were upregulated significantly at 1 dpi (Cornelissen et al., 2012). Here, the expressions of TLR3, TLR7, MDA5, and MAVS were clearly upregulated in the lungs and spleen of chickens inoculated with Q24 and Q26 H7N9 HPAIVs at 24 HPI. Thus, our results show that the H7N9 HPAIVs could activate host innate immune responses that are mediated by both TLRs and RLRs in chickens.

After the PRRs are activated, they can induce the secretion of type I interferons (IFNs) and pro-inflammatory cytokines. The type I IFNs could further trigger the expressions of ISGs to regulate host antiviral innate immune response and limit viral replication (Ranaware et al., 2016). However, over-expressed pro-inflammatory cytokine could cause an over-inflammatory response, which might result in destructive tissue inflammation and increase the host morbidity and mortality (Chi et al., 2013; Burggraaf et al., 2014). A previous study found that the expressions of IL-6, IL-8, and IFN-β were upregulated mildly in the lungs (1.14–1.51-fold) of chickens inoculated with chicken-origin H7N9 LPAIV in 2013 at 3 dpi (Jiao et al., 2018). Furthermore, the expressions of IFN-β, IFN-γ, IL-6, and IL-1β in the lungs, brain, and spleen of chickens infected by turkey-origin H7N1 HPAIV were significantly upregulated (Cornelissen et al., 2012). In this study, the expression levels of IL-6, IL-8, and IFN-β in the lungs and spleen of chickens inoculated with Q24 and Q26 H7N9 HPAIVs were also clearly upregulated at 24 HPI, and the IL-6 and IFN-β levels were upregulated in the lungs by 341.02–381.48 and 472.50–500.56-fold, respectively. Therefore, these data show that the H7N9 HPAIVs could efficiently activate innate immune responses and induce the expressions of type I IFNs and pro-inflammatory cytokines.

During virus infection, viral RNA activates host PRRs to trigger the signal pathway of type I IFNs, which induce the expressions of ISGs to result in the production of a series of antiviral proteins (such as OAS and Mx1), and thus produce the antiviral effect (Ranaware et al., 2016). Previous studies had shown that the expression of Mx1 was downregulated in the lungs but upregulated in the brains of chickens infected with chicken-origin H7N9 LPAIV at 3 dpi, and upregulated mildly in the lungs of chickens infected by duck-origin H9N2 LPAIV at 6 dpi (Ranaware et al., 2016; Jiao et al., 2018). Furthermore, the duck-origin H5N1 HPAIV induced excessive expressions of Mx1 and OAS in the lungs of infected chickens (Ranaware et al., 2016). Here, the expressions of Mx1 and OAS in the lungs and spleen of chickens inoculated with Q24 and Q26 H7N9 viruses were upregulated significantly at 24 HPI. Thus, Mx1 and OAS are also associated with resistance to the infection of H7N9 HPAIVs in chickens.

During virus infection, MHC I and II molecules are responsible for presenting antigen peptides produced by virus particles to CD8^+^ and CD4^+^ T cells to activate cellular immune response of the host (Neefjes et al., 2011). Our previous study found that the expression of MHC I was upregulated obviously in the brain and lungs of ducks inoculated with duck-origin H5N1 HPAIV (Wei et al., 2013). The expression of MHC I was upregulated significantly in the lungs of Pekin ducks infected with H5N1 HPAIV (Vanderven et al., 2012). Furthermore, the expression of MHC II was upregulated in the lungs of chickens infected with chicken-origin H6N2 LPAIV (Xing et al., 2008). In this study, the expressions of MHC I and MHC II in the lungs and spleen of chickens inoculated with Q24 and Q26 H7N9 viruses were upregulated significantly at 24 HPI. Thus, MHC I and MHC II were involved in regulating antiviral cellular immune responses induced by the H7N9 HPAIVs.

In conclusion, we have shown that the Q24, Q65, Q66, Q85, and Q102 H7N9 HPAIVs from Guangdong, southern China in 2017, clustered into two different genotypes. The Q24 and Q26 viruses replicated efficiently in chickens and were transmitted rapidly among them. The two HPAIVs led to severe clinical illnesses and deaths of chickens. The expressions of immune-related genes were upregulated significantly in the lungs and spleen of chickens infected by them at 24 HPI. These results provide insight into the evolution, pathogenic characteristics, and host antiviral immune responses of new H7N9 HPAIVs in chickens.

## Data availability statement

The datasets presented in this study can be found in online repositories. The names of the repository/repositories and accession number(s) can be found at: https://www.ncbi.nlm.nih.gov/genbank/, OP716876–OP716883; https://www.ncbi.nlm.nih.gov/genbank/, OP717067–OP717074; https://www.ncbi.nlm.nih.gov/genbank/, OP718194–OP718216; and https://www.ncbi.nlm.nih.gov/genbank/, OQ024903.

## Ethics statement

The animal study was reviewed and approved by all experiments involving animals were performed in ABSL-3 facilities and experimental protocols (SCAUABSL2017-004) were approved by the biosafety committee of South China Agriculture University. Housing animals were conducted in accordance with guidelines of the experimental animal administration and ethics committee of South China Agricultural University (SCAUABSL2017-004).

## Author contributions

BZ, WW, and PJ designed this study and performed the experiments. YS, XW, SF, WL, YD, ZC, and ZH assisted with experiment. BZ, WW, and PJ drafted the manuscript. SW, SF, and PJ participated in writing the discussion. All authors contributed to the article and approved the submitted version.

## Funding

This work was supported by grants from the National Key Research and Development Program of China (2021YFD1800202, 2016YFD0500207), the National Natural Science Foundation of China (32072844, 31872497), Laboratory of Lingnan Modern Agriculture Project (NT2021007), and the cooperation project of FAAS of China (DWHZ2021-02).

## Conflict of interest

The authors declare that the research was conducted in the absence of any commercial or financial relationships that could be construed as a potential conflict of interest.

## Publisher’s note

All claims expressed in this article are solely those of the authors and do not necessarily represent those of their affiliated organizations, or those of the publisher, the editors and the reviewers. Any product that may be evaluated in this article, or claim that may be made by its manufacturer, is not guaranteed or endorsed by the publisher.
